# Intraoperative Hyperglycemia May Be Associated with an Increased Risk of Myocardial Injury after Non-Cardiac Surgery in Diabetic Patients

**DOI:** 10.3390/jcm10225219

**Published:** 2021-11-09

**Authors:** Sojin Kim, Jungchan Park, Hara Kim, Kwangmo Yang, Jin-ho Choi, Kyunga Kim, Jidong Sung, Joonghyun Ahn, Seung-Hwa Lee

**Affiliations:** 1 Department of Anesthesiology and Pain Medicine, Samsung Medical Center, Sungkyunkwan University School of Medicine, Seoul 06351, Korea; sojin.kim@samsung.com (S.K.); jc83.park@samsung.com (J.P.); hara.kim@samsung.com (H.K.); 2 Department of Biomedical Sciences, Ajou University Graduate School of Medicine, Suwon 16499, Korea; 3 Center for Health Promotion, Samsung Medical Center, Sungkyunkwan University School of Medicine, Seoul 06351, Korea; kmhi.yang@samsung.com; 4 Department of Emergency Medicine, Samsung Medical Center, Sungkyunkwan University School of Medicine, Seoul 06351, Korea; jin-ho.choi@samsung.com; 5 Statistics and Data Center, Research Institute for Future Medicine, Samsung Medical Center, Seoul 06351, Korea; kyunga.j.kim@samsung.com (K.K.); jhguy.ahn@samsung.com (J.A.); 6 Department of Digital Health, SAIHST, Sungkyunkwan University, Seoul 06351, Korea; 7 Department of Cardiology, Rehabilitation & Prevention Center, Heart Vascular Stroke Institute, Samsung Medical Center, Sungkyunkwan University School of Medicine, Seoul 06351, Korea; jidong_sung@samsung.com; 8 Department of Biomedical Engineering, Seoul National University College of Medicine, Seoul 03080, Korea

**Keywords:** non-cardiac surgery, cardiac troponin, hyperglycemia

## Abstract

Background: Hyperglycemia in surgical candidates is associated with increased mortality and morbidity. We aimed to evaluate the effect of intraoperative blood glucose level on the incidence of myocardial injury after non-cardiac surgery (MINS) in diabetic patients. Methods: Diabetic patients with available intraoperative blood glucose measurement during non-cardiac surgery were enrolled in this study. Based on the highest intraoperative blood glucose level, patients were stratified into two groups: the blood sugar glucose (BST) < 180 group (intraoperative peak glucose < 180 mg/dL) and BST ≥ 180 group (intraoperative peak glucose ≥ 180 mg/dL). The primary outcome was the incidence of MINS, and secondary outcomes were in-hospital and 30-day mortalities. Results: Of the 11,302 diabetic patients, 8337 were in the BST < 180 group (73.8%) and 2965 in the BST ≥ 180 group (26.2%). After adjustment with inverse probability weighting, MINS was significantly higher in the BST ≥ 180 group (24.0% vs. 17.2%; odds ratio (OR), 1.26; 95% confidence interval (CI), 1.14–1.40; *p* < 0.001). In addition, in-hospital and 30-day mortalities were also higher in the BST ≥ 180 group compared to the BST < 180 group (4.2% vs. 2.3%, hazard ratio (HR), 1.39; 95% CI, 1.07–1.81; *p* = 0.001, and 3.1% vs. 1.8%; HR, 1.76; 95% CI, 1.31–2.36; *p* < 0.001, respectively). Receiver-operating characteristic plots showed that the threshold of glucose level associated with MINS was 149 mg/dL. Conclusion: Intraoperative hyperglycemia was associated with an increased MINS incidence and postoperative mortality in diabetic patients. Close monitoring of intraoperative blood glucose level may be helpful in detection and management of MINS.

## 1. Introduction

Hyperglycemia is associated with adverse outcomes in various clinical settings [1,2,3]. During surgical procedures, blood glucose level elevates as a normal physiological response to surgical stimuli regardless of diabetes status. However, diabetic patients suffer a higher risk of hyperglycemia owing to pathophysiologic changes such as impairment of vascular endothelium and cellular damage, leading to cardiovascular dysfunction, a major cause of mortality and morbidity [4,5]. Numerous studies have recommended an adequate control of blood glucose level in diabetic patients undergoing surgical procedures, but mainly in cardiac surgeries [6,7,8] or patients in critically ill states [9,10,11]. Therefore, there is a paucity of data on the association between intraoperative blood glucose level and cardiovascular events during non-cardiac surgical procedures.

Myocardial injury after non-cardiac surgery (MINS) is the most common cardiovascular complication in surgical patients, with an incidence of 8–18% and is regarded as an independent predictor of mortality [12,13]. Previously, MINS was shown to be associated with preoperative hyperglycemia [14,15]. However, considering that a transient elevation of glucose level in response to surgical stress is also associated with cardiovascular events [16], a separate analysis seems to be needed to assess intraoperative blood glucose level. Therefore, we aimed to evaluate whether intraoperative hyperglycemia is associated with the incidence of MINS in diabetic patients and estimated the cutoff value of the intraoperative peak blood glucose level associated with MINS. Our results may provide valuable evidence supporting the need for close monitoring of glucose levels during non-cardiac surgery in diabetic patients.

## 2. Methods

### 2.1. Study Population and Data Collection

In this retrospective observational study, we analyzed data from the SMC-TINCO registry (Samsung Medical Center Troponin in Non-cardiac Operation), under approval of the Institutional Review Board at Samsung Medical Center (SMC 2019-08-048), Seoul, Korea, and the same committee waived the requirement for informed written consent. Before patient enrollment, cohort registration was conducted at https://cris.nih.go.kr (accessed on 26 August 2019) (KCT0004244). The present study was conducted in accordance with the precepts of the Declaration of Helsinki and results are reported regarding the “Strengthening the Reporting of Observational Studies in Epidemiology” guidelines [17]. This registry used de-identified data from the institutional electronic research data repository, Clinical Data Warehouse Darwin-C, and consists of 43,019 consecutive patients who had cardiac troponin (cTn I) levels measured before or within 30 days after non-cardiac surgery between 2010 and 2019 at Samsung Medical Center. Demographic and perioperative data including medical, surgical, and anesthetic records were obtained from the registry. Mortality statistics were derived from the documents, and deaths outside of our institution were updated and verified with the National Population Registry of the Korea National Statistical Office.

The exclusion criteria for this study were as follows: (1) patients younger than 18 years old at the time of surgery, (2) patients with no postoperative cTn I measurements, (3) patients who required chest compression before the diagnosis of MINS, and (4) patients without available intraoperative blood glucose measurements. A total of 11,302 diabetic patients were included in the final analysis to examine the effect of transient hyperglycemia on diabetic patients.

Patients with a history of diabetes in their medical records or who used anti-diabetic medications at the time of admission were classified as diabetic. These patients were stratified according to elevation in intraoperative glucose level, ≥180 mg/dL, which was obtained from arterial blood gas analysis during surgery [18,19,20,21].

### 2.2. Study Outcomes and Definitions

The primary outcome was incidence of MINS, defined as a peak cTn I level above the 99th percentile of the upper reference limit (URL) within 30 days after surgery. Following the diagnostic criteria, a non-ischemic etiology that shows elevations of cTn I, such as pulmonary embolism, sepsis, cardioversion, atrial fibrillation, or chronic elevation of cTn I, was not considered MINS [12,13]. Active cancer was defined as a histologic confirmation of malignancy within six months before surgery [22]. High-risk surgery was grouped with a reported mortality risk greater than 5%, according to the 2014 European Society of Cardiology/European Society of Anesthesiology (ESC/ESA) guidelines [23]. The secondary outcomes were in-hospital mortality and 30-day mortality.

### 2.3. Perioperative cTn I Measurement and Management

At our hospital, cTn I measurement is recommended for patients with at least one of the following major cardiovascular risk factors: history of heart failure, ischemic heart disease, diabetes on insulin therapy, stroke, or chronic kidney disease. In patients with minor risk factors, cTn I was measured at the discretion of the attending clinician, considering recently suspected symptoms of ischemic disease or advanced age. It was also recommended for those undergoing moderate- to high-risk surgery. An automated analyzer (Advia Centaur XP; Siemens Healthcare Diagnostics, Erlangen, Germany) with a highly sensitive cTn I immunoassay was used. According to the manufacturer, the lowest limit of detection was 6 ng/L, and the 99th percentile URL was 40 ng/L [24]. Patients with elevated cTn I received consultation by a cardiologist and specialized management by an attending clinician.

### 2.4. Intraoperative Glucose Measurement and Management

Patients undergoing surgery followed a strict nil per os; nothing by mouth (NPO) protocol for at least 8 h of fasting from fatty food or meat before surgery. Therefore, the sole marker for glycemic status, which reflects acute fluctuation during the intraoperative period, was the fasting glucose. For patients with uncontrolled blood glucose elevation prior to surgery, endocrinology consultation was arranged. Oral anti-glycemic drugs were withheld on the morning of surgery and restarted after surgery, when oral intake was permitted.

The management of diabetic patients followed the National Health Service (NHS) [18] and several other guidelines [19,20,21]. We intended to maintain blood glucose level below 180 mg/dL (10 mmol/L), and when significant hyperglycemia (blood glucose >180 mg/dL) was detected, regular insulin was infused continuously or loaded as bolus injections at the discretion of an attending anesthesiologist. Glucose level was measured in arterial blood from a preplaced line with a blood gas analyzer (RAPIDLab 1200 Blood Gas Analyzer; Siemens Healthcare, Erlangen, Germany) at 37 °C. An arterial line was indicated if hemodynamic instability or significant bleeding was anticipated. In addition to its role in continuous monitoring of invasive blood pressure and beat-to-beat variation in the patient’s cardiovascular system, blood samples could be drawn repeatedly from this peripheral arterial line.

### 2.5. Statistical Analysis

Differences were compared by the Mann–Whitney test or Student’s *t*-test for continuous data and presented as the median values with interquartile range or mean ± standard deviation. Categorical data are presented as number (%), and Fisher’s exact test or chi-square was applied to examine any association between categorical variables. The stratified logistic regression model was used to compare the incidence of MINS, and was reported as an adjusted odds ratio (OR) with a 95% confidence interval (CI). Cox regression analysis was used to compare mortalities and reported as hazard ratio (HR) with 95% CI.

To adjust for potential selection bias and imbalance in patient baseline characteristics, we employed an inverse probability weighted Cox regression model (IPW) [25]. To observe any interaction among relevant covariates, a subgroup analysis was conducted and presented as a forest plot. Pearson’s correlation coefficient and receiver-operating characteristic (ROC) plots were conducted to assess the predictability of intraoperative glucose threshold in predicting MINS. The statistical power based on the sample size was calculated using Spearman’s rank correlation. Sensitivity analyses of unmeasured confounders were performed. Statistical analyses were performed with R 4.0.2 (Vienna, Austria; http://www.R-project.org/ (accessed on 10 February 2021)) using the PowerSurvEpi (0.1.3) and Sensuc (6.2-0) packages (Boston, USA: https://cran.r-project.org/web/packages/powerSurvEpi/index.html (accessed on 10 February 2021)). All data were evaluated using two-tailed *t*-tests, and statistical significance was assumed at *p*-values < 0.05.

## 3. Results

**Figure 1:**
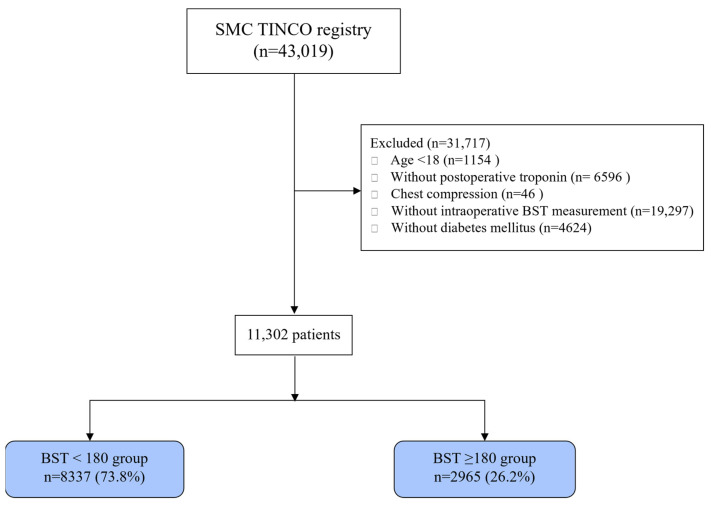
Flowchart of study patients.

Among 43,019 patients in the registry, the exclusion criteria were as follows: 19,297 patients without measurement of intraoperative glucose levels, 6596 patients without postoperative cTn I measurements, 1154 patients younger than 18 years old at the time of surgery, and 46 patients who required chest compression prior to the diagnosis of MINS. The final study population consisted of 11,302 diabetic patients with available intraoperative blood glucose levels (Figure 1).

Patients were divided into two groups according to the intraoperative glucose level: 8337 patients (73.8%) with peak blood glucose level < 180 mg/dL (BST < 180 group), and 2965 patients (26.2%) with peak blood glucose level ≥ 180 mg/dL (BST ≥ 180 group) (Table 1). Intraoperative peak glucose level > 180 mg/dL was considered as significant hyperglycemia. Patients in the BST ≥ 180 group tended to be more hypertensive, current alcoholics, and older, and tended to have active cancer, a history of preoperative in-hospital insulin administration, extracorporeal membranous oxygenation, ventilator, and continuous renal replacement therapy. The percentage use of intraoperative insulin was higher in the BST ≥ 180 group. Differences were seen in operative variables of ESC/ESA operation risk, emergency operation requirement, operation duration, and need for continuous infusion of inotropic agents or transfusion of packed red blood cells. The peak troponin levels in the BST < 180 group and the BST ≥ 180 group were 0.54 (±8.3) and 1.02 (±12.2), respectively (*p* = 0.018). The percentage of patients with available preoperative C-reactive protein (CRP) was 83% and 76.2% in the BST < 180 group and BST ≥ 180 group, and the values were 1.62 (±4.42) and 2.02 (±4.6), respectively (*p* < 0.001). The preoperative pro-brain natriuretic peptide (BNP) measurement was performed in 22% of patients in the BST < 180 group and 25.4% of patients in the BST ≥ 180 group, and their values were 1584.1 (±2539.1) and 1628.5 (±5336.5), respectively (*p* = 0.846). Preoperative use of anti-hyperglycemic and cardiac medication is listed in Table 2. The types of surgery each group had undergone are listed in Supplemental Table S1.

**Figure 2:**
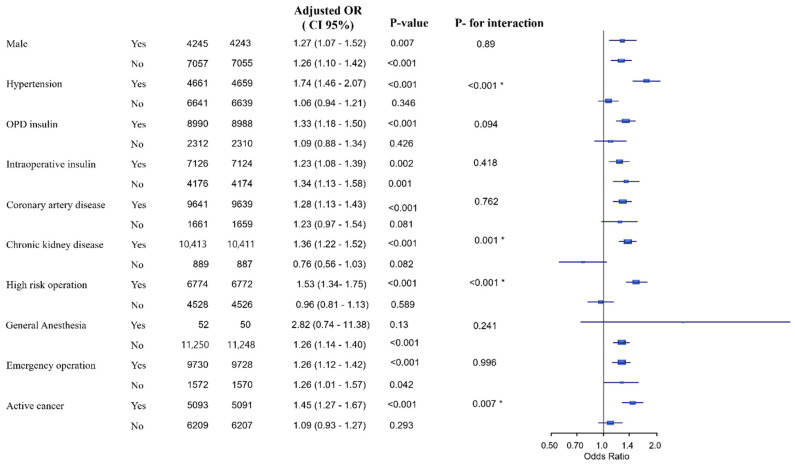
Forest plot from subgroup analysis showing the association between significant hyperglycemia and risk of MINS. * *p*- for interactions for these variables are significant (*p* < 0.005).

**Figure 3:**
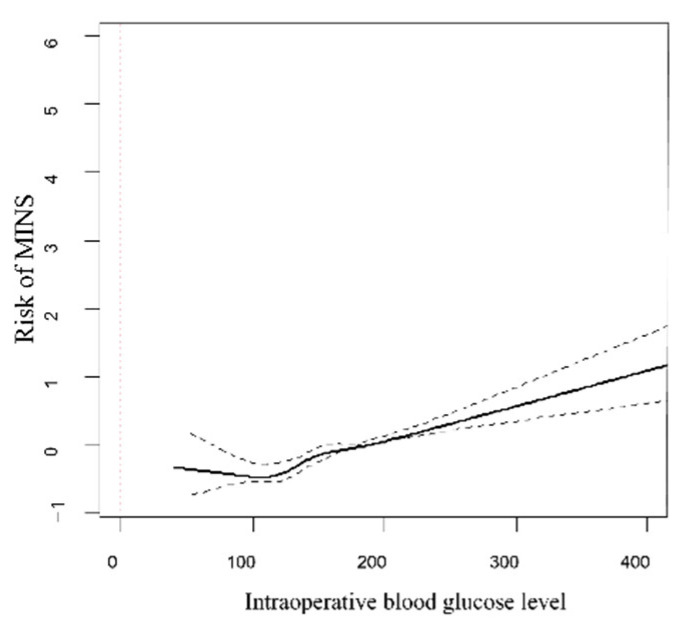
Spline curve for the log odds ratio of intraoperative blood glucose level versus risk of MINS.

**Figure 4:**
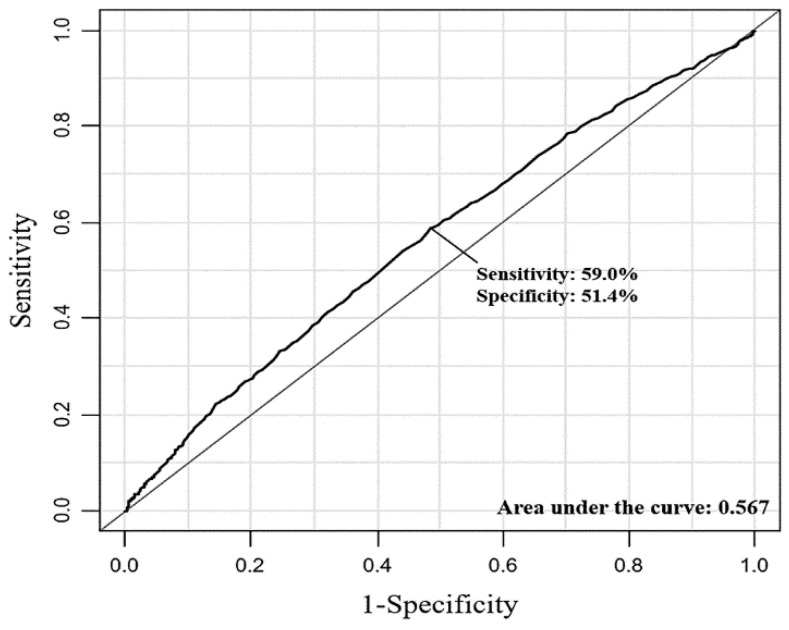
Receiver-operating characteristic plots to estimate the threshold of intraoperative blood glucose level associated with MINS.

Of the 3193 patients with postoperative cTn elevation, 110 were diagnosed with a non-ischemic cause; therefore, a total of 2147 (19.0%) patients were diagnosed with MINS. The incidence of MINS was significantly higher in the BST ≥180 group after adjustment with multivariable analysis (24.0% vs. 17.2%; OR, 1.34; 95% CI, 1.20–1.50; *p <* 0.001) (Table 3). Our results remained significant after IPW adjustment, and the incidence of MINS was significantly associated with hyperglycemia (OR, 1.30; 95% CI, 1.17–1.44; *p <* 0.001) (Table 3). Our study also revealed significantly higher risk of in-hospital mortality and 30-day mortality in the BST ≥180 group (4.2% vs. 2.3%; adjusted OR, 1.45; 95% CI, 1.14–1.83; *p* < 0.001, and 3.1% vs. 1.8%; adjusted OR, 1.94; 95% CI, 1.48–2.56; *p <* 0.001, respectively) (Table 3). These results were consistent after IPW adjustment (OR, 1.34; 95% CI, 1.02–1.75; *p =* 0.01 for in-hospital mortality and OR, 1.78; 95% CI, 1.33–2.38; *p <* 0.001 for 30-day mortality, respectively). Postoperative diagnosis of MINS patients is shown in Table 4. Subgroup analysis revealed that the association between hyperglycemia and MINS was confounded by hypertension, chronic renal failure, high-risk surgical operation, and active cancer (*p* for interaction <0.001, *p <* 0.001, *p =* 0.001, and *p =* 0.007, respectively) (Figure 2). The incidence of MINS was significantly increased by hyperglycemia only in patients without hypertension (OR 1.74; 95% CI, 1.46–2.07, *p <* 0.001), without chronic renal failure (OR 1.36; 95% CI, 1.22–1.52, *p =* 0.001), without active cancer (OR 1.45; 95% CI, 1.27–1.67, *p <* 0.001), and who were not undergoing a high-risk operation (OR 1.53; 95% CI, 1.34–1.75, *p <* 0.001). The spline curve for the log odds ratio of intraoperative blood glucose level versus risk of MINS was plotted (Figure 3). The estimated threshold for intraoperative glucose level associated with MINS was 149 mg/dL, according to ROC analysis, and the area under the ROC curve was 0.57. The sensitivity and specificity were 59.0% and 51.4%, respectively (Figure 4). The power analysis of the sample size was 0.99 when the OR for MINS was 1.3 and 0.95 when the OR for MINS was 1.2. The effect of an unmeasured confounder was estimated with an assumed prevalence of 40%, and the observed associations were all significant (Supplemental Table S2).

## 4. Discussion

This study demonstrated that significant hyperglycemia in diabetic patients during non-cardiac surgery confers an increased MINS and mortality incidence. The estimated threshold for intraoperative blood glucose level associated with MINS was 149 mg/dL. Our findings suggest that monitoring intraoperative blood glucose level may be beneficial for early detection and management of MINS.

Perioperative hyperglycemia is common as a result of transient body glucose elevation in response to physiological stress among patients undergoing surgery. The stress of surgery and anesthesia causes the release of counter-regulatory hormones, leading to reduced insulin production and increased lipolysis [4]. Sustained hyperglycemia triggers insulin resistance by impairment of the insulin signal transduction pathway, resulting in cardiovascular events such as myocardial injury [14,26]. Perioperative hyperglycemia is also associated with numerous other adverse outcomes including longer postoperative hospital duration, surgical site infection, higher overall morbidity, and mortality [1,27]. Inducing acute changes in cellular metabolism, such as an increase in polyol pathway activation and hexosamine pathway flux, activation of protein kinase C, formation of advanced glycation end products, and hyperglycemia, leads to tissue damage, vascular inflammation, fibrosis, and injury by overproduction of reactive oxygen species [28].

Our study focused on the effect of transient hyperglycemia (BST ≥ 180 mg/dL) superimposed on diabetic patients at risk for various adverse clinical outcomes and complications related to diabetes itself. According to our result, intraoperative hyperglycemia was associated with an increased risk of MINS. This result is consistent with our previous finding that MINS was associated with preoperative glucose level rather than hemoglobin A1c level, which reflects more of a long-term glucose status [15]. Both results stress the association between short-term glycemic monitoring and incidence of MINS. Since blood glucose level is a significant and independent mortality predictor among diabetic patients who already experience a higher risk of cardiovascular disease, close surveillance and assessment of diabetic patients during and after surgery are indeed necessary.

The mediation of reactive oxygen species could explain the mechanism behind the association between hyperglycemia and MINS. The imbalance between free radical generation and elimination plays a key role in the pathophysiology of myocardial injury [28]. During the perioperative period, oxidative stress due to hyperglycemia and insulin resistance contributes to the cellular structure and functions of diabetic myocardium [29,30]. The number of myocyte precursor cells that are responsible for differentiation of cardiac immature cells to mature myocyte and cell proliferation was reduced due to high levels of oxygen reactive species in hyperglycemic patients with acute myocardial infarction (AMI) [31]. Furthermore, surgical hypercoagulability, bleeding, and inflammation can predispose patients to ischemic injuries [32].

In this study, we divided the patients according to intraoperative blood glucose levels of 180 mg/dL, while the estimated threshold associated with MINS incidence was 149 mg/dL. Stress hyperglycemia has been related consistently to adverse outcomes in both diabetic and non-diabetic patients [27,33]. In a previous study, even modest glucose elevation (140–179 mg/dL) was strongly related to mortality only in non-diabetic patients, but more severe hyperglycemia (glucose > 180 mg/dL) was a risk factor for mortality among diabetic patients [2]. Because the purpose of this study was to evaluate the effect of stress-induced hyperglycemia in diabetic patients, we stratified patients into the hyperglycemia group even with a single intraoperative glucose level over 180 mg/dL. Therefore, our results need to be validated in patients without diabetes. Of note, the subgroup analysis showed that the observed association between hyperglycemia and MINS was not significant in patients with hypertension, chronic renal failure, active cancer, and those undergoing high-risk surgery. In addition, the threshold of intraoperative blood glucose associated with MIN had an area under the ROC curve of 0.57 with relatively low sensitivity and specificity; therefore, further investigation regarding a more precise cutoff value for intraoperative blood glucose level is required.

For intraoperative blood glucose control, the 2019 American Diabetes Association guidelines recommend a range between 80–180 mg/dL (4.4–10.0 mmol/L) [34], based on numerous studies suggesting tight glycemic control during surgery [6,35]. Tight glycemic control was associated with significant improvement of the regenerative potential of the ischemic myocardium in patients with AMI [31]. According to 2017 European Society of Cardiology guidelines for management of acute myocardial infarction in patients with ST segment elevation, glucose-lowering therapy should be initiated in acute coronary syndrome patients with glucose levels >180 mg/dL [36]. Another study in type 2 diabetic patients with cardiovascular risks showed that intensive glycemic control resulted in a reduction of high sensitivity C-reactive protein [37]. On the other hand, conflicting results have been reported, suggesting that aggressive intraoperative treatment of hyperglycemia did not improve outcome; in some instances, such treatment actually increased mortality and postoperative stroke [16,30]. While the optimal intraoperative treatment algorithm to lower blood glucose when undergoing non-cardiac surgery is still controversial, preventing hypoglycemia (glucose < 70 mg/dL) is emphasized in many literatures [34,36,38].

For the patients of the present study, regular insulin was used to treat hyperglycemia during the surgical period. The use of regular insulin is known to have anti-inflammatory, anti-thrombotic, and anti-atherogenic properties with a favorable effect on the myocardium [39,40]. However, an accurate blood glucose target during surgery was not identified in our study and needs further investigation.

There are several limitations to be addressed. Due to the nature of a retrospective study, perioperative cTn I and intraoperative blood glucose levels were not measured in all patients undergoing non-cardiac surgery; as they were selectively performed in high-risk patients due to the institutional protocol, our results might have been exaggerated by selection bias, and may not be generalizable to all patients. Although efforts were made to adjust for confounding factors, there still remains a possibility that the results were affected by unmeasured covariates. Moreover, a unifying treatment protocol for perioperative hyperglycemic management was absent. Anesthesia was provided by many different anesthesiologists; therefore, a therapeutic approach to hyperglycemia such as the dose of insulin given and at which glucose level the treatment initiation has taken place can vary, possibly influencing the results. Lastly, our study could not fully address the question of adequate blood glucose level or whether treatment of hyperglycemia with insulin may reduce the incidence of MINS or mortality. Despite these limitations, this study is the first to demonstrate that intraoperative hyperglycemia was associated with the incidence of MINS and mortality in diabetic patients, highlighting the importance of glycemic control during surgical procedures. We believe that this study may provide valuable guidance for further research.

## 5. Conclusions

Intraoperative hyperglycemia (fasting glucose ≥ 180 mg/dL) appears to be associated with an increased risk of MINS in diabetic patients. Continuous monitoring and management of intraoperative glucose level may potentially protect diabetic patients from developing MINS. Intensified risk assessment of diabetic patients with hyperglycemia during the perioperative period may also alert clinicians as to early detection and intervention of adverse cardiovascular events. Further studies are needed to confirm our findings.

## Institutional Review Board Statement

The present study was conducted in accordance with the precepts of the Declaration of Helsinki and approved by the Institutional Review Board at Samsung Medical Center (SMC 2019-08-048), Seoul, Korea.

## Figures and Tables

**Table 1:** Baseline characteristics according to intraoperative blood glucose level.

	BST < 180	BST ≥ 180	Before IPW		After IPW	
	(*n* = 8337)	(*n* = 2965)	*p*-Value	SMD	*p*-Value	SMD
Intraoperative minimum BST *	120.3 (25.8)	168.8 (44.2)	<0.001		<0.001	
Intraoperative maximum BST *	134.8 (25.6)	215.2 (35.8)	<0.001		<0.001	
Intraoperative insulin, units *	21.7 (±39.3)	29.1 (±265.9)	0.013		0.651	
Intraoperative insulin use	2925 (35.1)	1251 (42.2)	<0.001	14.6%	0.270	2.5%
Age	61.0 (±13.7)	63.4 (±11.2)	<0.001	19.8%	0.046	4.7%
Male	5235 (62.8)	1822 (61.5)	0.203	2.8%	0.243	2.7%
Hypertension	4733 (56.8)	1908 (64.4)	<0.001	15.6%	0.428	1.9%
Chronic kidney disease	637 (7.6)	252 (8.5)	0.147	3.2%	0.832	0.5%
Current alcoholic	1753 (21.0)	515 (17.4)	<0.001	9.3%	0.917	0.3%
Active cancer	4408 (52.9)	1801 (60.7)	<0.001	15.9%	0.505	1.6%
Previous disease						
Stroke	573 (6.9)	196 (6.6)	0.656	1.0%	0.891	0.3%
Coronary artery disease	1191 (14.3)	470 (15.9)	0.042	4.4%	0.641	1.1%
Heart failure	194 (2.3)	43 (1.5)	0.005	6.4%	0.298	2.6%
Arrhythmia	559 (6.7)	160 (5.4)	0.014	5.5%	0.218	3.2%
Heart valvular disease	81 (1.0)	19 (0.6)	0.124	3.7%	0.644	1.3%
Preoperative in-hospital care						
Insulin use	7508 (90.1)	2788 (94.0)	<0.001	14.7%	0.026	5.9%
Intensive care unit	390 (4.7)	205 (6.9)	<0.001	9.6%	0.004	6.6%
ECMO	1 (0.0)	0 (0.0)	>0.999	1.5%	0.554	1.4%
Continuous renal replacement therapy	41 (0.5)	18 (0.6)	0.549	1.6%	0.177	2.9%
Ventilator	58 (0.7)	44 (1.5)	<0.001	7.6%	0.007	5.9%
Operative variables						
ESC/ESA high-risk operation	2950 (35.4)	1578 (53.2)	<0.001	36.5%	0.334	2.2%
General anesthesia	8289 (99.4)	2961 (99.9)	0.004	7.4%	0.817	0.9%
Emergency operation	1100 (13.2)	472 (15.9)	<0.001	7.7%	0.211	2.9%
Operation duration	4.13 (±2.12)	4.99 (±2.31)	<0.001	38.7%	0.422	1.8%
Continuous infusion of inotropics	3097 (37.1)	1221 (41.2)	<0.001	8.3%	0.713	0.8%
Packed red blood cell transfusion	1048 (12.6)	561 (18.9)	<0.001	17.5%	0.255	2.5%

Data are presented as *n* (%), mean (± standard deviation), or median (interquartile range); IPW, inverse probability weighting; SMD, standardized mean difference; BST, blood sugar test; ECMO, extracorporeal membranous oxygenation; ESC, European Society of Cardiology; ESA, European Society of Anaesthesiology. * These variables were not retained in the IPW.

**Table 2:** Preoperative medical treatment.

	BST < 180	BST ≥ 180	
	(*n* = 8337)	(*n* = 2965)	*p*-Value
Insulin	1728 (20.7)	617 (21.0)	0.69
Metformin	1617 (19.4)	1183 (39.9)	<0.001
Beta-blocker	1753 (21.0)	638 (21.5)	0.592
Calcium channel blocker	2522 (30.3)	1093 (36.9)	<0.001
ACE-inhibitor	546 (6.5)	209 (7.0)	0.37
ARB	2561 (30.7)	1080 (36.4)	<0.001
Statin	2414 (29.0)	979 (33.0)	<0.001
Aspirin	2027 (24.3)	724 (24.4)	0.93
Clopidogrel	655 (7.9)	225 (7.6)	0.67
Warfarin	312 (3.7)	81 (2.7)	0.012
RAAS	2817 (33.8)	1184 (39.9)	<0.001
Anti-platelet	2492 (29.9)	902 (30.4)	0.60
DOAC	94 (1.1)	28 (0.9)	0.47

Data are presented as *n* (%), mean (±standard deviation) or median (interquartile range).

**Table 3:** The incidence of myocardial after non-cardiac surgery and mortality according to preoperative blood glucose level.

	BST < 180 (*n* = 8337)	BST ≥ 180 (*n* = 2965)	Univariable Analysis		Multivariable Analysis		After IPW	
			Unadjusted OR/HR (95% CI)	*p*-Value	Adjusted OR/HR * (95% CI)	*p*-Value	OR/HR (95% CI)	*p*-Value
MINS	1434 (17.2)	713 (24.0)	1.52 (1.38–1.69)	<0.001	1.34 (1.20–1.50)	<0.001	1.30 (1.17–1.44)	<0.001
In-hospital mortality	191 (2.3)	125 (4.2)	1.39 (1.11–1.74)	0.005	1.45 (1.14–1.83)	<0.001	1.34 (1.02–1.75)	0.01
30-day mortality	147 (1.8)	93 (3.1)	1.80 (1.39–2.33)	<0.001	1.94 (1.48–2.56)	<0.001	1.78 (1.33–2.38)	<0.001

Data are presented as *n* (%), mean (±standard deviation), or median (interquartile range).* Covariates include age, hypertension, history of active cancer, preoperative use of metformin, insulin, intraoperative red blood cell transfusion, operation duration, and high-risk operation.

**Table 4:** Postoperative diagnosis in MINS patients.

	BST < 180	BST ≥ 180	*p*-Value
	(*n* = 1437)	(*n* = 713)	
Postoperative Diagnosis			
Myocardial infarction	31 (0.4)	24 (0.8)	0.005
ST-elevation	7 (0.1)	1 (0.0)	0.630
Non-ST-elevation	24 (0.3)	23 (0.8)	0.001

Data are presented as *n* (%), mean (±standard deviation), or median (interquartile range).

## Data Availability

Not applicable.

## Supplementary Materials

The following are available online at https://www.mdpi.com/article/10.3390/jcm10225219/s1, Table S1: Types of surgery. Table S2: Sensitivity analysis of the effect of an unmeasured confounder on odds ratio for incidence of myocardial injury after non-cardiac surgery due to intraoperative hyperglycemia.
