# Supplementary material for: Intraoperative Hyperglycemia May Be Associated with an Increased Risk of Myocardial Injury after Non-Cardiac Surgery in Diabetic Patients
Source: J Clin Med. 2021 Nov 9;10(22):5219. doi: 10.3390/jcm10225219 (PMC8623971; doi:10.3390/jcm10225219)
Supplement: Supplementary file 1 [file jcm-10-05219-s001.zip › jcm-1438518-supplementary.pdf]

Journal of
Clinical Medicine

MDPI

Intraoperative Hyperglycemia May Be Associated with an Increased Risk of Myocardial Injury after Non-Cardiac Surgery in Diabetic Patients

Sojin Kim [1,†], Jungchan Park [1,2,†], Hara Kim [1], Kwangmo Yang [3], Jin-ho Choi [4], Kyunga Kim [5,6], Jidong Sung [7], Joonghyun Ahn [5] and Seung-Hwa Lee [7,8,*]

[1] Department of Anesthesiology and Pain Medicine, Samsung Medical Center, Sungkyunkwan University School of Medicine, Seoul 06351, Korea; sojin.kim@samsung.com (S.K.); jc83.park@samsung.com (J.P.); hara.kim@samsung.com (H.K.)
[2] Department of Biomedical Sciences, Ajou University Graduate School of Medicine, Suwon 16499, Korea
[3] Center for Health Promotion, Samsung Medical Center, Sungkyunkwan University School of Medicine, Seoul 06351, Korea; kmhi.yang@samsung.com (K.Y.)
[4] Department of Emergency Medicine, Samsung Medical Center, Sungkyunkwan University School of Medicine, Seoul 06351, Korea; jin-ho.choi@samsung.com (J.-h.C.)
[5] Statistics and Data Center, Research Institute for Future Medicine, Samsung Medical Center, Seoul 06351, Korea; kyunga.j.kim@samsung.com (K.K.); jhguy.ahn@samsung.com (J.A.)
[6] Department of Digital Health, SAIHST, Sungkyunkwan University, Seoul 06351, Korea
[7] Department of Cardiology, Rehabilitation & Prevention Center, Heart Vascular Stroke Institute, Samsung Medical Center, Sungkyunkwan University School of Medicine, Seoul 06351, Korea; jidong_sung@samsung.com (J.D.S.)
[8] Department of Biomedical Engineering, Seoul National University College of Medicine, Seoul 03080, Korea
* Correspondence: shuaaa.lee@samsung.com; Tel: +82-2-3410-3214; Fax: +82-2-3410-3849
† The first two authors contributed equally to this work (S.K., J.P.).

Supplemental Table S1. Types of surgery.

	**BST < 180 (*n*=8337)**	**BST ≥ 180 (*n*=2965)**
Vascular	490 (5.9)	166 (5.6)
Orthopediatric	299 (3.6)	88 (3.0)
Neuro	2335 (28.0)	357 (12.0)
Breast or Endo	96 (1.2)	28 (0.9)
Plastic or Otolaryngeal or Eye	108 (1.3)	44 (1.5)
Transplantation	607 (7.3)	332 (11.2)
Gynecology or Urology	265 (3.2)	134 (4.5)
Gastrointestinal	3346 (40.1)	1575 (53.1)
Noncardiac thoracic	781 (9.4)	236 (8.0)
Others	10 (0.1)	5 (0.2)

Supplemental Table S2. Sensitivity analysis of the effect of an unmeasured confounder on odd ratio for incidence of myocardial injury after non-cardiac surgery due to intraoperative hyperglycemia.

		**$OR_{ZY\|X}$**					
		**1.5**	**2**	**2.5**	**3**	**3.5**	**4**
	0.3	1.77 (1.59 - 1.97)	1.96 (1.76 - 2.19)	2.14 (1.92 - 2.39)	2.31 (2.06 - 2.58)	2.48 (2.21 - 2.78)	2.64 (2.35 - 2.96)
	0.4	1.70 (1.53 - 1.89)	1.84 (1.65 - 2.05)	1.96 (1.76 - 2.19)	2.08 (1.87 - 2.32)	2.19 (1.96 - 2.45)	2.30 (2.05 - 2.57)
OR_{zx}	0.5	1.65 (1.49 - 1.84)	1.75 (1.58 - 1.95)	1.84 (1.66 - 2.05)	1.93 (1.73 - 2.15)	2.01 (1.80 - 2.24)	2.08 (1.86 - 2.32)
	0.6	1.62 (1.46 - 1.79)	1.69 (1.52 - 1.88)	1.76 (1.58 - 1.95)	1.82 (1.63 - 2.02)	1.87 (1.68 - 2.09)	1.93 (1.73 - 2.15)
	0.7	1.59 (1.43 - 1.76)	1.64 (1.48 - 1.82)	1.69(1.52 - 1.87)	1.73 (1.56 - 1.92)	1.77 (1.59 - 1.97)	1.81 (1.62 - 2.01)

Prevalence of unmeasured confounder = 40%; Numbers represent ORs. (including 95% CIs); OR, odds ratio; HR, hazard ratio; X: dichotomous exposure measure, y dichotomous outcome measure, z: potential dichotomous confounder; ORZX indicates the association (OR) between the unmeasured confounder and intraoperative hyperglycemia; ORZY|X indicates the association (OR) between the unmeasured confounder and incidence of MINS conditional on exposure status.
